# Dynamic changes in the secondary structure of ECE-1 and XCE account for their different substrate specificities

**DOI:** 10.1186/1471-2105-13-285

**Published:** 2012-11-01

**Authors:** Zaheer Ul-Haq, Sadaf Iqbal, Syed Tarique Moin

**Affiliations:** 1 Dr. Panjwani Center for Molecular Medicine and Drug Research, International Center for Chemical and Biological Sciences, University of Karachi, Karachi, 75270, Pakistan

## Abstract

**Background:**

X-converting enzyme (XCE) involved in nervous control of respiration, is a member of the M13 family of zinc peptidases, for which no natural substrate has been identified yet. In contrast, it’s well characterized homologue endothelin-converting enzyme-1 (ECE-1) showed broad substrate specificity and acts as endopeptidase as well as dipeptidase. To explore the structural differences between XCE and ECE-1, homology model of XCE was built using the complex structure of ECE-1 with phosphoramidon (pdb-id: 3DWB) as template. Phosphoramidon was docked into the binding site of XCE whereas phosphate oxygen of the inhibitor was used as water molecule to design the apo forms of both enzymes. Molecular dynamics simulation of both enzymes was performed to analyze the dynamic nature of their active site residues in the absence and presence of the inhibitor.

**Results:**

Homology model of XCE explained the role of non-conserved residues of its S2’ subsite. Molecular dynamics (MD) simulations identified the flexible transitions of F149/I150, N566/N571, W714/W719, and R145/R723 residues of ECE-1/XCE for the strong binding of the inhibitor. Secondary structure calculations using DSSP method reveals the folding of R145/R723 residue of ECE-1/XCE into β-sheet structure while unfolding of the S2’ subsite residues in aECE-1 and sustained compact folding of that of aXCE. The results evaluated are in good agreement with available experimental data, thus providing detailed molecular models which can explain the structural and specificities differences between both zinc peptidases.

**Conclusions:**

Secondary structure changes of both enzymes during the simulation time revealed the importance of β-sheet structure of R145/R723 for its binding with the terminal carboxylate group of the inhibitor. Unfolding of the α-helix comprising the S2’ subsite residues in aECE-1 correlate well with its endopeptidase activity while their compact folding in aXCE may account for the inactivity of the enzyme towards large C-terminal containing substrates.

## Background

Zinc peptidases such as matrix metalloproteinases (MMPs) [1,2] angiotensin converting enzyme (ACE) [3,4] and neutral endopeptidase (NEP) [5] are involved in peptide metabolism. The peptide metabolism is activated by degradation of a wide range of bioactive peptides and therefore specific inhibitors of them have therapeutic values [6,7]. One of the important classes of M13 family (of zinc peptidases; classification according to MEROPS database) [8] is “gluzincins” which is defined by a HExxH motif including two histidines and a glutamic acid as zinc-coordinating ligands. Zinc peptidases of neprilysin family are gluzincins that include several enzymes for instance, neutral endopeptidase (NEP) [9], NEP2 [10]/soluble secreted endopeptidase (SEP) [11]/neprilysin-like enzyme 1 (NL1) [12]/membrane metalloendopeptidase-like 2 (MMEL2) [13], endothelin-converting enzymes ECE-1 and ECE-2 [14,15], the KELL blood group protein [16], the phosphate-regulating neutral endopeptidase on the X chromosome (PHEX) [17], and X-converting enzyme (XCE) [18]/endothelin-converting enzyme-like 1 (ECEL1) [19] /rodent homologue damaged-induced neuronal endopeptidase (DINE) [20].

XCE (nowadays are known as ECEL1 but we used XCE in this manuscript to differentiate with ECE-1) is expressed in the nervous system, particularly in the medulla oblongata and in the spinal cord, presumably by cholinergic neurons such as motor neurons or striatum interneurons. The physiological function of XCE was first reported from the inactivation of the corresponding gene in mice, which described the enzyme as to play a vital role in the nervous control of respiration [21]. Benoit *et al*. found that XCE is mostly located in the cellular endoplasmic reticulum (ER) consistent with the high mol. wt smears observed on SDS-PAGE, while only less than 10% portion of the enzyme reaches the cell surface. They also suggested the enzyme’s function in both compartments i.e., ER and the cell surface [22]. ER is considered as a target organelle for XCE to suppress stress due to responding of the enzyme to nerve injury [23]. The presence of XCE at the cell surface refers one of the enzyme’s functions to the regulation of the activity of extracellular peptides like other family members such as ECE-1 and NEP performed. Several candidate neuropeptides such as endothelin, galanin, calcitonin, bradykinin, met- and leu-enkephalins, and somatostatin were considered as potential substrates for XCE activity. Particularly, galanin was supposed to be the most probable one because of its presence and degradation in spinal cord/cerebrospinal fluid. Inhibition of the galanin degradation was reported to be done only by phosphoramidon [24], but none other inhibitors were found active against XCE [18]. The enzyme was found active only against a synthetic tripeptide substrate [20], but so far, no obvious reason of the inactivity of XCE against potential neuropeptides has been reported in the literature inspite of they linked its subcellular localization with the lack of success in the identification of extracellular substrate. More work is therefore needed to identify its extracellular substrates to answer questions concerning the enzyme’s precise function.

Contrary to XCE, its well studied homologue ECE-1 can efficiently cleaves variety of substrates. ECE-1 exists as a disulfide-linked homodimer *invivo* and cleaves the W21-V22 bond in big endothelin-1 (ET-1), a potent vasoconstrictor [14]. The monomeric C412S mutant of rat ECE-1 (C428S in human) has been shown to have much lower efficiency for the cleavage of big ET-1 as compare to the wild type showing dimerization of ECE-1 which is preferred for effective conversion of big ET-1 into ET-1 [25]. Furthermore, M. V. Hoang and A. J. Turner established that ECE-1 also cleaves the unrelated bradykinins (BK) at a significant rate, in addition to its *in-vivo* substrate big ET-1 (endopeptidase action), thereby acting as a peptidyl dipeptidase. The lack of sequence similarity in the BK peptides and the peptidyl dipeptidase revealed broad specificity and additional physiological roles for ECE-1 possibly linked to its subcellular location [26]. Moreover, recombinant ECE-1 was found to have minimal activity against small substrates (smaller than hexapeptides), such as Leu-enkephalin. However, large peptides such as neurotensin, substance P, bradykinin, and the oxidized insulin B chain were also observed to be hydrolyzed by the enzyme as efficiently as the big ET-1 [27] was. In spite of various natural substrates known upto date, the detailed mechanism of the cleavage of the substrates for such a variable length and unrelated sequences is still lacking for ECE-1. Hence, the understanding of extracellular substrate activities of ECE-1 and XCE remain challenging despite of their several activity profiling experiments because of the lack of appropriate structural knowledge.

Because of the difficulties encountered in the crystallization of proteins specially of the membrane proteins, homology modeling is being served as a valuable tool since last two decades to solve the three-dimensional structures of proteins having at least one X-ray crystal structure of homologous protein [28,29]. For neprilysin family, initially crystal structure knowledge of thermolysin, a bacterial protein, was used to model the structural features of these proteins. Crystal structure of neutral endopeptidase (pdb-id: 1DMT), the only well characterized member opened new door to acquire structural knowledge of several members of this class of the protein [30]. The three-dimensional structure of ECE-1 was also solved with the co-crystallized metalloprotease inhibitor phosphoramidon (pdb-id: 3DWB) [31]. XCE shared much identity with ECE-1 and was also identified as ECE-like protein, therefore the crystal structure coordinates of ECE-1 was utilized to rationalize the structural facts of XCE. Here, the three-dimensional structures of XCE with and without the metalloprotease inhibitor i.e., phosphoramidon were modelled. However, a molecular-level understanding of the function of a biological macromolecule requires knowledge of both its structural and dynamical properties. The dynamical flexibility of both proteins in their inhibitor bound and unbound forms were explored by molecular dynamics (MD) simulations. Our aim is to identify the structural differences between the modeled XCE and ECE-1 by elucidating conformational flexibility of both the enzymes. The characterization of structure and dynamics of both proteins and in particular their subsites with respect to phosphoramidon would be significant to understand the structural differences responsible for their different substrate specificities and to design specific inhibitors against both zinc peptidases.

## Results and discussion

### Homology modeling

In the absence of experimental structures, computational methods are used to predict 3D protein models to provide insight into the structure and function of proteins. There are several successfull examples where homology modeling has aided in the prediction of protein function [32-35]. However, the choice of template, inaccurate alignments and inefficient refinement methods are still the main sources of errors in homology modeling [36]. We therefore critically checked the modeling of XCE at every step from template alignment to refinement before validating the final model from external sources.

#### Sequence conservation between both proteins

**Figure 1:**
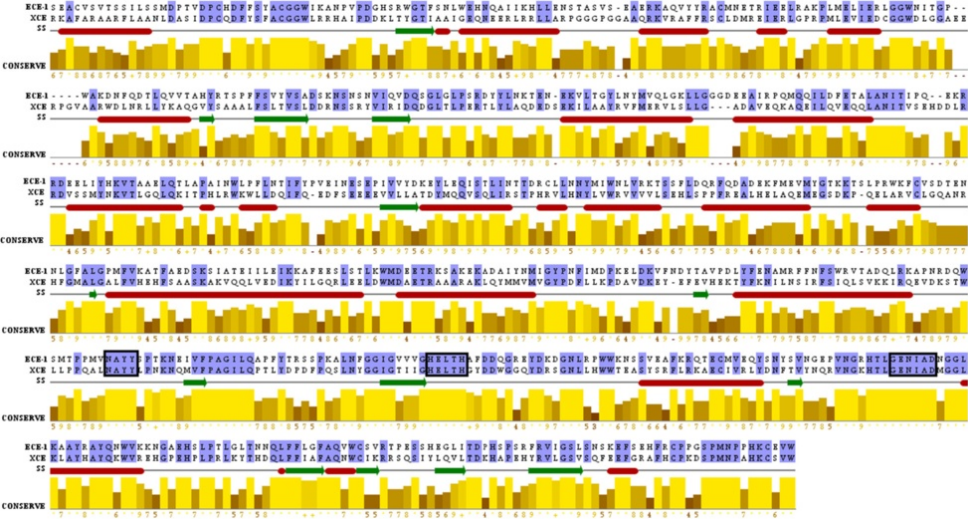
**Primary sequence alignment between XCE and ECE-1.** Jalview is used for alignment. Signature of zinc peptidases are highlighted in rectangle boxes. Identical residues are marked with dot whereas high conservation in the amino acid sequences of both proteins is shown in bright yellow color and higher numerical value.

Primary sequence alignment showed that amino acid residues 1–100 in XCE could not be modeled due to lack of equivalent residues in ECE-1 structure. Therefore, R102 is considered as the first amino acid of the ectodomain of XCE model which is aligned with S101 of ECE-1 as depicted from Figure 1. Final alignment comprised of 4 gaps in XCE sequence, and these gaps are not longer than 3 amino acids. Three characteristic motifs, 565VNAYY569, 607HELTH611, and 667ENIAD671 of ECE-1, hallmarks of the M13 family of zinc peptidases, are conserved and correspond to 570LNAYY574, 612HELTH616, and 672ENIAD676 motifs in XCE respectively. The presence of VNAYY motif in ECE-1 is responsible for the specificity towards big endothelins binding whereas semi-conserved LNAYY motif in XCE may have influence on the binding of substrate [37]. Two histidines H612 and H616 in the HELTH motif and one glutamate E672 in the ENIAD motif coordinate to the catalytic zinc (Zn^2+^) in XCE. Corresponding to E608 of ECE-1, E613 of XCE is believed to act as a nucleophile which promotes the attack of zinc-bound water molecule on the scissile peptide. The resulting transition state formed during hydrolysis of substrate gets stabilized by H737 in XCE corresponding to H732 of ECE-1. D614 and D671 of ECE-1 form Asp-His-Zinc triad to place zinc atom appropriately for catalysis and identical role can also be predicted for the conserved D619 and D676 in XCE. Finally, the evaluation of stereochemical properties is presented by the Ramachandran plot provided as Additional file 1: Figure S1.

#### Comparison between protein-inhibitor complexes

**Figure 2:**
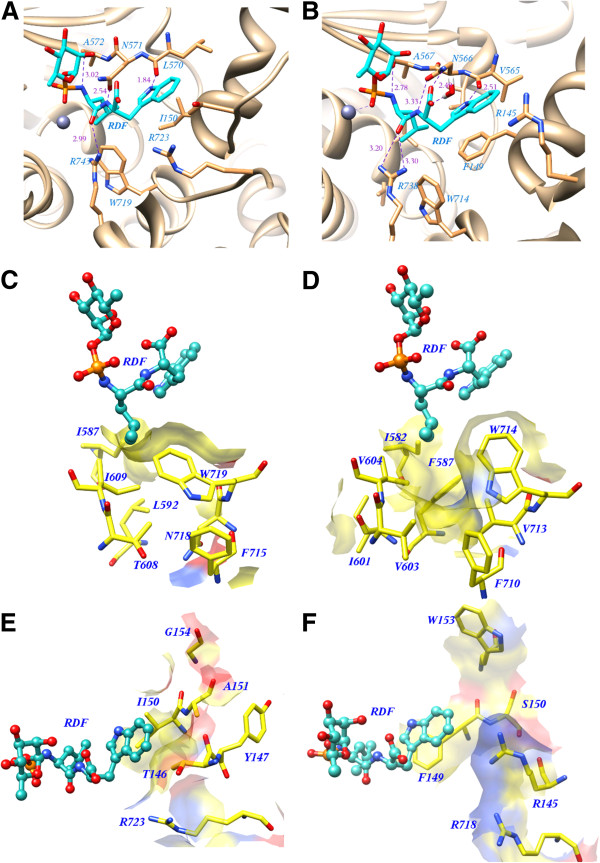
**Comparison of hydrogen bonding and subsites residues.** Main chain hydrogen bonding interactions of the inhibitor phosphoramidon with (**A**) XCE and (**B**) ECE-1. (**C**) Amino acid residues of the S1’ pocket of XCE and (**D**) ECE-1. (**E**) Amino acid residues of the S2’ pocket of XCE and (**F**) ECE-1. Hydrophobic surfaces are shown in yellow, positively changed areas in blue and negatively charged surfaces in red.Ligand phosphoamidon is shown in ball and stick model whreas amino acids of the pocket are depicted in sticks.

The docked conformation of phosphoramidon in the modelled XCE is compared in terms of its interaction with the X-ray bound conformation of ECE-1. Figure 2A represents the docked conformation of phosphoramidon in complex with XCE whereas the X-ray bound conformation of phosphoramidon with ECE-1 is shown in Figure 2B. The plausibility of the enzyme inhibitor complex in zinc peptidases is primarily strengthened by the backbone hydrogen bond contributors from “NAYY” motif. The carbonyl oxygen of A572 in XCE created a weak hydrogen bond with the P1’ amide nitrogen of phosphoramidon in a similar manner as formed by A567 in ECE-1. Two hydrogen bonds were found for N566 of ECE-1 with the P2’ amide nitrogen and oxygen atoms of the terminal carboxylate while its equivalent N571 of XCE maintained only one H-bond with the P2’ amide nitrogen. In XCE, however, V565 of ECE-1 was replaced with L570 amino acid, but hydrogen bonds between the P2’ indole moiety of phosphoramidon and their carbonyl oxygen atoms are conserved in both proteins. R743 of XCE was also involved in the hydrogen bond with the P1’ carbonyl oxygen of the inhibitor as observed for R738 of ECE-1. Hence, all hydrogen bonding interactions are also found in the XCE-bound docked conformation of the inhibitor.

The specificity of all metalloproteases depends on the interactions of the P1’ and P2’ moieties of their substrates/inhibitors with the corresponding S1’ and S2’ subsites of the enzyme. We used the well characterized subsite specificity information of ECE-1 to understand the structural difference in XCE present at the subsite level. The amino acid residues I587, L592, I606, T608, I609, F715, N718 and W719 constitute the S1’ subsite in XCE corresponding to the large pocket of ECE-1 comprising I582, F587, I601, V603, V604, F710, V713 and W714 residues as illustrated from Figures 2C and 2D, respectively. The P1’ group with large hydrophobic side-chains indicates the specificity of ECE-1 due to the large hydrophobic S1’ subsite. The presence of non-conserved hydrophilic T608 in XCE corresponding to V603 of ECE-1 did not change the overall hydrophobic environment of the S1’ recognition pocket because of the formation of strong hydrogen bond with G578 located outside the binding pocket. Replacement of V604 with I609 in XCE provided more hydrophobic environment than that of ECE-1, thus resulting in a favorable interaction with the P1’ hydrophobic side-chain of the inhibitor. N718 in XCE occupied more space which increases the depth and flexibility of the S1’ subsite in XCE as compared to ECE-1 with small V713.

The S2’ subsites of XCE and ECE-1 contain all non-conserved amino acids except one arginine as shown in Figures 2E and 2F, respectively. The side-chain of R145, S150, and W153 in ECE-1 occupied more space and volume than their counterpart T146, A151, and G154 residues in XCE. Schulz *et al.*[31] suggested that the sidechains of the S2’ subsite residues in ECE-1 were disordered and did not establish any hydrogen bond due to which, indole moiety of phosphoramidon was not appropriately anchored into the S2’ pocket of ECE-1. On the other hand, all residues of the S2’ subsite in XCE are found very close, thus leading to very narrow binding pocket responsible for strong binding of phosphoramidon at this region. R145 of ECE-1 formed water mediated hydrogen bonds with the terminal carboxylate of phosphoramidon, which are lacking in XCE due to the non-conserved T146 residue. Table 1 summarizes the effect of non-conserved subsite residues of both enzymes and their possible consequences on the inhibitor binding. The binding pattern of phosphoramidon and its relative stability into the active site of both proteins are further explored with the help of MD simulations.

**Table 1:** Consequence of non-conserved amino acid residues of XCE

**ECE-1**	**XCE**	**Change in Nature**	**Structural features**	**Subsite**
R145	T146	Basic to nucleophilic	No binding with carboxylate	S2’
W146	Y147	Aromaticity conserved	Volume of subsite is shrinked	S2’
F149	I150	Aromatic to aliphatic	Aromatic wall is absent	S1’
S150	A151	Nucleophilic to hydrophobic	No overall change	S2’
W153	G154	Aromatic to small aliphatic	Volume of subsite is shrinked	S2’
V565	L570	Hydrophobicity conserved	H-bond acceptor is conserved	NAYY
F587	L592	Aromatic to aliphatic	Aromatic wall is absent	S1’
V603	T608	Hydrophobic to nucleophilic	No overall change	S1’
V604	I609	Hydrophobicity conserved	More interaction with the P1’ moiety	S1’
V713	N718	Hydrophobic to hydrophilic	Polarity at the bottom of subsite	S1’

### Molecular dynamics simulations

Molecular dynamics simulations permit characterization of biomolecular processes such as the conformational transitions associated with the functions of protein at molecular and atomic level. Nowadays, this technique is being extensively used to study the conformational flexibility of biological macromolecules because of the advances in computer hardware, software, and MD algorithms [38]. We calculated fluctuations in protein structure for all four systems aXCE, cXCE, aECE-1, cECE-1 by means of nanosecond timescale MD simulations which are proved to be a complementary technique for experiments. Modern computer architectures provide means to perform MD simulations of varying time length that can range from nanoseconds to microsecond-timescale or even at millisecond timescale. However, long timescale MD simulations based on all-atom force fields are still not so convincing to be employed. Since most of the force fields were parameterized previously and are therefore not extensively applied in this time regime. As an illustration of the problems related to unrealistic and irreversible structural changes, the DNA simulation of the timescale beyond 20–30 ns time could be exemplified [39]. Such long timescale MD simulations of biological macromolecules utilizing enhanced sampling methods and/or coarse-graining methods were frequently reported. On the other hand short timescale MD simulations such as of few nanoseconds have serious concerns about dealing the process of relaxation and the proper sampling of the equilibrium dynamics. These two significant factors must be kept in mind when presenting results based on the short MD run. Therefore, to ensure about sufficient amount of sampling which is a hard task, the use of replicate simulations could be helpful to address this issue. In the absence of replicate simulations the situation becomes worse to test proper sampling as well as the reproducibility of the results obtained from the simulations could be challenged. Could be an alternative of the replicate simulations, the use of better analysis of the equilibration process would be significant. Plotting root mean square deviation (RMSD) from the time-averaged structure (rmsd_time−averaged_) calculated from the simulation trajectories of all systems would also adequately answer other relevant questions about sampling time as opposed to rmsd_init_.

#### Root mean square deviation (RMSD) of the protein backbone

**Figure 3:**
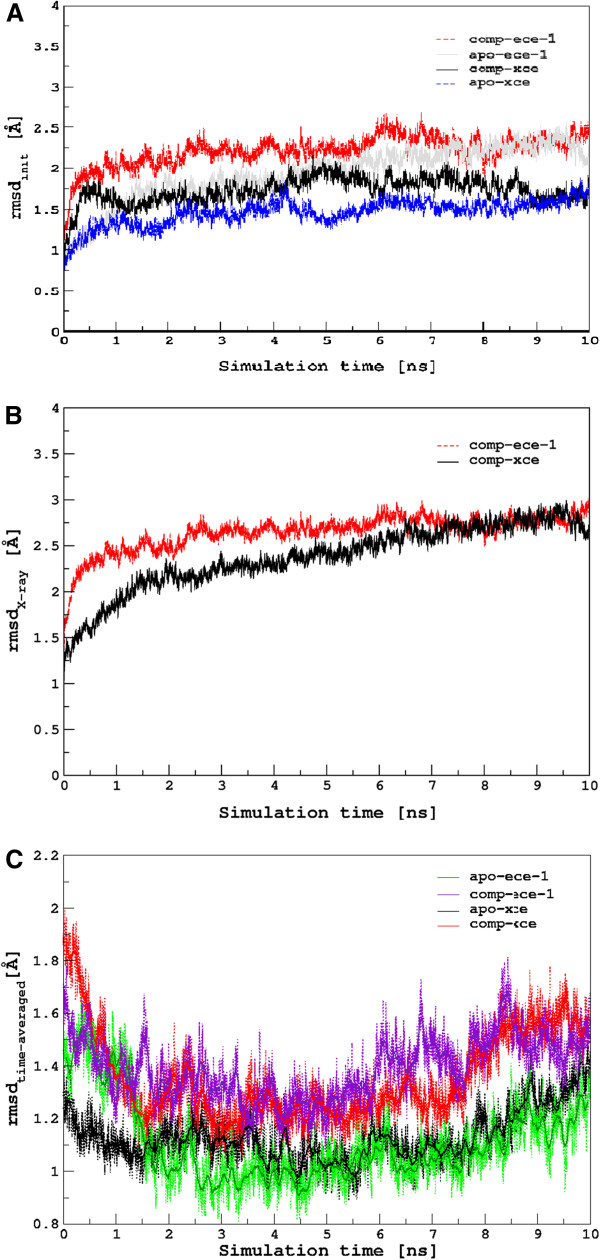
**Comparison between root mean square deviations.** Root mean square deviations calculated over a time course of 10 ns for (**A**) αC backbone atoms of the four simulated systems (black) apo XCE, (blue) complexed XCE, (grey) apo ECE 1, and (red) complexed ECE-1 (**B**) the heavy atoms of cECE-1 and cXCE as a reference of their X-ray and the modeled structures, respectively **(C)** the backbone atoms of the four simulated systems with respected to time averaged structures.

An initial evaluation involves analysis of the protein structure via root mean square deviations (RMSD) of backbone atoms with respect to the equilibrated structure (rmsd_init_) to perform sampling of trajectories and to evaluate the stability of proteins throughout the simulation time as illustrated in Figure 3A. The rmsd_init_ for apo XCE (aXCE) fluctuated between 1.0 and 1.8 Å with an average value of 1.45 Å whereas rmsd_init_ of the inhibitor bound XCE (cXCE) appeared from the same starting point as in aXCE but after 1 ns it exhibited a large deviation up to 2.5 Å, which indicates changes in the protein structure due to protein-inhibitor complex formation. The template protein ECE-1 in their apo (aECE-1) and complexed form (cECE-1) showed an increased rmsd_init_ value due to more flexibility compared to its homologue XCE in their corresponding forms (aXCE and cXCE). The complex formation in ECE (cECE-1) resulted in rmsd_init_ difference of ~0.48 Å from the average rmsd_init_ value calculated for its apo form (~1.72 Å). The difference in rmsd_init_ values for aXCE and cXCE resembles to ECE-1 proteins which indicates the similar conformational changes in both proteins from the binding of phosphoramidon which will be described in latter sections. Figure 3B illustrates the root mean square deviation with respect to the X-ray structure of all heavy atoms of ECE-1 which exhibited a fluctuation between ~2.5 and ~3.0 Å with an average value of 2.6 Å. The identical approach was used to analyze the structural deviation in terms of rmsd with respect to the initial homology model of XCE (Figure 3B), which varied between ~2.0 and ~3.0 Å, thus showing a significant structural deviation from the homologue structure. Besides these two rmsd plots such as rmsd_init_ and rmsd with respect to X-ray structure which are considered poor metrics to observe equilibration process, the rmsd_time−averaged_ plots of all proteins forming V-shape could therefore be a fair substitute to observe the under-sampled simulations. The smallest rmsd values in the plots shown in Figure 3C are concentrated around the center of the simulations of 10 ns showing that the equilibration is in progress. U-shaped rmsd_time−averaged_ plots were obtained but not V-shaped that show that the equilibration is in progress along with some contaminations of relaxation processes. Nevertheless, the behavior shown by the simulations having both equilibration and a small amount relaxation processes can still be regarded as providing biologically significant information. A question can also arisen that why the short MD run could provide a conformation close to that of the native state of the protein as determined via X-ray structure (pdb-id : 3DWB) [31]. This issue was addressed by Ramachandran plots (Additional file 2: Figure S2, Additional file 3: Figure S3, Additional file 4: Figure S4, Additional file 5: Figure S5) developed for all the equilibrated structures that are in excellent agreement with experimental data. This validates the simulation approach which was used to yield the nearly native conformation of the protein as all simulations adopted the near crystal structure distributions with some variation in the secondary structure elements. The approach is therefore, applicable to analyze other properties of the given size of protein.

#### B-factor and secondary structure calculations

**Figure 4:**
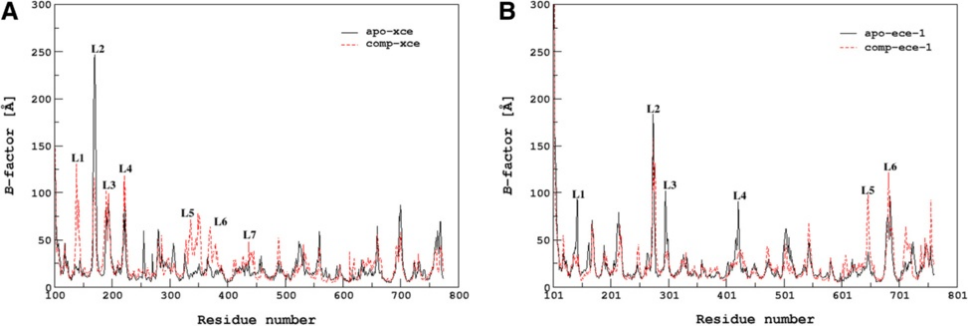
**Comparison of B-factors between apo and complexed forms.** The calculated B-factor for the 10 ns simulation of the apo and complexed forms of (**A**) XCE and (**B**) ECE-1 proteins.

**Figure 5:**
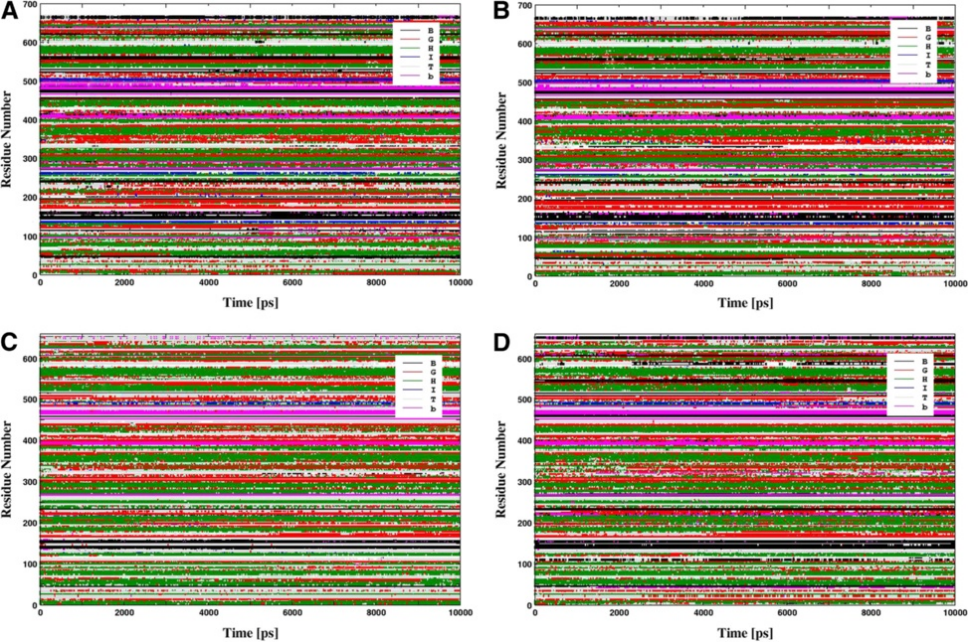
**Secondary structure changes in both proteins.** The calculated secondary structure changes by DSSP method for (**A**) apo XCE (**B**) complexed XCE (**C**) apo ECE-1 and (**D**) complexed ECE-1. Color labels are used as black (B - antiparallel β-sheet), grey (T - turn), green (H - α-helix), magenta (b - parallel β-sheet), red (G - 3-10 helix), and blue (I - π-helix).

The fluctuations in the local structure of XCE and ECE-1 protein in their apo and complexed forms were further investigated employing the *B*-factors (*Bi*) calculation for αC atom of each residue. Figure 4A and 4B depict calculated *B*-factors of (apo and complexed) XCE and ECE-1, respectively reflecting significant fluctuations in the loop regions. The calculated average *B*-factor of cECE-1 (24.3 Å^2^) was lower than average crystallographic *B*-factors for αC atom (49.3 Å^2^) resulting from a higher degree of hydration in the simulation compared to the crystal structure. Considering the information obtained from comparison between theoretical and experimental *B*-factors of cECE-1 protein, the similar approach was used to calculate average *B*-factors of each XCE amino acids. The average cXCE *B*-factors was calculated as 24.18 Å^2^ which is comparable to the average *B*-factor of cECE-1 indicating a bit lower dynamic flexibility of cXCE compared to cECE-1. Secondary structure calculations were also performed to correlate fluctuations of residues with the corresponding changes in the secondary structure elements for aXCE and cXCE, as shown in Figures 5A and 5B, respectively. Amino acid residues 137–147 of aXCE located in the region L1 comprised mostly of loops along with a turn which underwent antiparallel β sheet conformation due to complexation with the inhibitor (cXCE). In aXCE, residues 145–146 were folded into β strand after a period of 4 ns whereas β strand of 143–145 residues in cXCE was degraded into loops during the simulation observed via visual inspection. Furthermore, amino acid residues 151–154 of the S2’ subsite passed from α-helix to 3–10 helix, which in turn affects the region L1 of XCE because it is found at the immediate vicinity of the S2’ subsite. Loop area L1 in cXCE was more fluctuated than that of aXCE, which indicates conformational modifications of this region after the inhibitor binding (Figure 4A). The region L2 (166–173) of both XCE comprised of a loop (166–170) and an α-helix (171–173), respectively. Secondary structure plots of XCE proteins (Figures 5A and 5B) suggest that the unfolding of the α-helix (171–173) results in an increased fluctuation of the region L2 in aXCE which is reduced upon binding with the inhibitor. Moreover, regions L3 (residues 190–194) and L4 (residues 221–224) are situated at far bottom of the active site and exhibited high level of fluctuations in the secondary structure from α-helix to 3-helix/turn and from 3–10 helix to loop/turn, respectively (Figures 5A and 5B). At the front of the binding site, most significant regions L5 (residues 330–350), L6 (residues 368–383) and L7 (residues 410–445) are comprised of loops only and therefore, are expected to show high flexibility. These regions exhibited more fluctuations in cXCE compared to aXCE indicating the direct influence of the inhibitor binding (Figure 4A). Major deviations in the secondary structure of the residues 330–338 of L5, 378–383 of L6, and 424–426 of L7 are observed as they are folded into α-helix in aXCE whereas they showed random transitions from 3–10 helix/turns to unstructured loops in cXCE (Figures 5A and 5B). Figure 4B illustrates *B*-factors of each amino acid residue of apo and complexed ECE-1 correlating the extent of structural modifications produced by the inhibitor. During simulation, the region L1 (residues 143–155) including residues of the S2’ subsite experiences changes in its secondary structure elements for both forms of ECE-1 as shown in Figures 5C and 5D. Amino acid residues 143–144 are present as turn in aECE-1 whereas as parallel β-sheet in cECE-1. R145 belonging to the S2’ subsite was folded into antiparallel β-sheet at initial stage of the simulation in both forms of ECE-1. It is suggested that the folding of R145 is necessary for its strong interaction with the terminal carboxylate. The conformation adopted by amino acid residues 150–155 in aECE-1 was a compact α-helix but during simulation this α-helix was transformed into a turn due to its drifting from the active site (visual inspection), however, it is found as a compact α-helix throughout the simulation in cECE-1 (Figures 5C and 5D). Amino acid residues 274–275 close to the active site constitute a loop (L2) which showed large fluctuations in both forms of ECE-1. In cECE-1, L3 (residues 296–297) and L4 (residues 421–428) get stabilized after the inhibitor binding attributed by their low fluctuations in B-factors while L5 (residues 646–647) and L6 (residues 681–691) showed higher fluctuations compared to those of aECE-1 (Figure 4B). Secondary structure calculations revealed conformational transformations into turns/β sheet/α-helix in the L4 region only whereas L3, L5, and L6 regions remain unstructured as turns/loops in both forms of ECE-1 (Figures 5C and 5D). Thus, inhibitor binding accounts for high fluctuations of the loop regions and affects overall secondary structure elements surrounding the active site of both XCE and ECE-1 proteins.

#### Conformational changes in the active site amino acids of XCE

**Figure 6:**
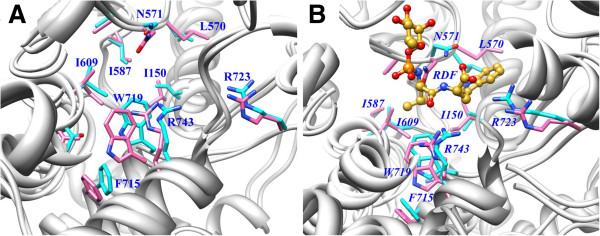
**Conformational changes in apo and complexed XCE.** Supeimposed coordinates of before (pink sticks) and averaged over 10 ns simulation (cyan sticks) for (**A**) apo and (**B**) complexed forms of XCE.

The conformational changes induced by the inhibitor on the active site of zinc peptidases have not been understood yet in detail due to the nonavailability of their 3D apo structures. The separate sampling of the bound and unbound states is the key to describe conformational differences upon binding. Figure 6A shows the superimposed aXCE structures before and averaged over 10 ns simulation whereas similar coordinates for cXCE are displayed in Figure 6B. Significant conformational changes in the side-chains of I150, L570, N571 and R723 are observed in both forms as a result of simulation. During simulation, the side-chain of I150 acquired cis conformation in cXCE, being close to the P1’ leucyl moiety of the inhibitor, whereas its trans conformation is stable in aXCE. N571 rotated its side-chain towards the P2’ carbonyl oxygen in cXCE during simulation. The inside bending of N571 is found initially in both forms of XCE while its outside bending is only observed during the simulation in cXCE which results in the formation of hydrogen bond (Figure 6B). Inside and outside transition of N571 drastically affects the hydrogen bonding interaction of L570 with the P2’ indole moiety of the inhibitor. All residues of the S1’ and S2’ subsites are stable in their initial position, however, W719 approached more close to the P1’ leucyl moiety of the inhibitor during simulation. The positively charged R723 in cXCE acquired extended conformation and attracted towards the negatively charged carboxylate of the inhibitor as the simulation proceed. DSSP calculations also revealed the folding of R723 into antiparallel β-sheet during simulation.

#### Conformational changes in the active site amino acids of ECE-1

**Figure 7:**
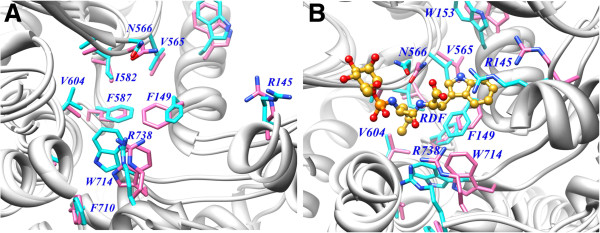
**Conformational changes in apo and complexed ECE-1.** Superimposed coordinates of before (pink sticks) and averaged over 10 ns simulation (cyan sticks) for (**A**) apo and (**B**) complexed forms of ECE-1.

Figure 7A depicts the active site residues of the superimposed aECE-1 structures before and averaged over 10 ns simulation while similar coordinates for cECE-1 are shown in Figure 7B. In cECE-1, the side-chain of R145 approached very close to the carboxylate group of the inhibitor during simulation, however, it was found far apart before simulation. DSSP calculations also illustrated the folding of R145 into antiparallel β-sheet during simulation. Side-chain nitrogen atom of N566 rotated towards the P1’ amidic NH group of the inhibitor in cECE-1 during simulation causes loss of its interaction which was found in the crystal structure (Figure 7B). Before simulation, F149, F587, and W714 are perpendicularly inclined towards the P1’ leucyl moiety of the inhibitor and known to form an aromatic wall which can block its deeper approach into the S1’ pocket. During simulation, W714 folded into 3–10 helix and approached more closer to the P1’ group of the inhibitor in cECE-1 as compared to the crystal structure. All residues of the S1’ subsite are stable in their initial position but I582 changes trans conformation in cECE-1. In aECE-1, remarkable conformational drift in the S2’ residues (149–153) from the active site is observed (Figure 7A), however, in cECE-1 this drifting is not found due to the presence of the inhibitor which can hold them towards itself. It is not identified yet that in order to fit into the interior of the enzyme whether big endothelins, the in-vivo substrates of ECE-1, forces an opening of the enzyme wall or requires a folded conformation of their C-terminal part [40]. Hence, the conformational drift in the S2’ residues identified in our simulation study correlates well with the activity of aECE-1 towards the large C-terminals containing natural substrates.

#### Stability of enzyme-inhibitor interactions

Both enzymes contain tetrahedral zinc ion (Zn^2+^) coordinated with H607/H612, H611/H616, E667/E672 and the PO1 atom of phosphoramidon in ECE-1/XCE, respectively. Table 2 summarizes the average coordinate bond distances whereas average hydrogen bond and hydrophobic interactions evaluated over stable period of 2–8 ns for the enzyme-inhibitor complexes are given in Table 3. The stability of the hydrogen bonds between the inhibitor and protein are probed by their percent occupancy. Backbone hydrogen bonding residues of XCE are L570, N571, and A572, which are equivalent to V565, N566, and A567 of ECE-1. The P1’ amidic nitrogen, the P2’ amidic nitrogen and the carbonyl oxygen of phosphoramidon strongly bound to the carbonyl oxygen of A572, the carbonyl oxygen and amidic nitrogen of N571 in XCE, also attributed by their high percent occupancy, whereas the sustainability of these interactions from A567 and N566 of ECE-1 is low (Table 3). During simulation, non-conserved residues L570 of XCE and V565 of ECE-1 got changes in their backbone dihedral angles which led to the loss of their interaction with the NH group of the indole moiety of phosphoramidon. Thus, it was analyzed that not even a single backbone residue of ECE-1 maintain its vital hydrogen bonding, which is also corroborated by the inhibitory potency of phosphoramidon in μM range against ECE-1. An hydrogen bond from R743 of XCE with the P1’ carbonyl oxygen showed very low occupancy compared to the corresponding bond from R738 of ECE-1 which comparatively attained higher stability (Table 3). I582 and V604 of ECE-1 remained close to the P1’ group during simulation, however, equivalent residues I587 and I609 of XCE were found far apart before simulation and only I609 established hydrophobic interaction during simulation (Table 3). F149 of ECE-1 formed van der Waal contact and π-π stacking to the P2’ indole moiety while I150 of XCE did not interact with the P2’ group but prefered hydrophobic interaction with the P1’ moiety due to its cis conformation (Table 3). Before simulation, R145 of ECE-1 was involved in water mediated hydrogen bonds with the terminal carboxylate but after 1500 ps these interactions are converted into strong salt bridge. Johnson *et al*. determined carboxydipeptidase activity of ECE-1 during the cleavage of bradykinin and substance P revealing that these substrates with COOH-terminal carboxylates are highly preferred over their cognate amides and esters [41]. Interestingly, formation of salt bridge from R145 coorelate with the carboxydipeptidase activity of ECE-1, however, Johnson *et al*. did not find any significant decrease in activity when measured against R133L mutant enzyme [41]. The responsibility of the terminal carboxylate binding in XCE is taken by R723 (equivalent to R718 of ECE-1) and in doing so, N571 and R723 of XCE behave like the terminal carboxylate binding residues N542 and R102 of neutral endopeptidase (NEP) [30]. During simulation, the binding mode of phosphoramidon in XCE is found similar to its binding in NEP and it can be speculated that phosphoramidon binds firmly with XCE just like its strong binding to NEP. The different binding conformation of phosphoramidon in XCE and ECE-1 strengthens our hypothesis that although XCE is more close to ECE-1 but still minor differences in the behavior of their equivalent residues of the active site and its surrounding affect their tendency towards natural substrates.

**Table 2:** Stability of zinc coordinating ligands throughout the simulations

**ECE-1 Residues**	**X-ray Dist. (Å)**	**Avg Dist. (Å) ± St. Dev**	**XCE Residues**	**Model Dist. (Å)**	**Avg Dist. (Å) ± St. Dev**
H607: NE2	2.24	2.06 ± 0.00	H607: NE2	2.06	2.03 ± 0.04
H611: NE2	2.13	2.03 ± 0.04	H611: NE2	2.05	2.05 ± 0.04
E667: OE1	1.75	1.95 ± 0.03	E667: OE1	1.95	1.95 ± 0.03
PO1	2.07	2.04 ± 0.07	PO1	1.86	2.01 ± 0.09
PO2	2.54	2.22 ± 0.13	PO2	3.69	2.25 ± 0.15

**Table 3:** Comparison between enzyme-inhibitor interactions and their stability

**Interacting atoms**			**Dist. before simulation (Å)**		**Avg. Dist. (Å) ± St. Dev (% occupancy)**	
**Inhibitor**	**ECE-1**	**XCE**	**ECE-1**	**XCE**	**ECE-1**	**XCE**
PO1	H732: NE2	H737: NE2	2.69	3.28	2.83 ± 0.13 (85.24)	2.79 ± 0.11 (88.10)
PO2	E608: OE2	E613: OE2	2.49	2.36	2.69 ± 0.13 (99.93)	2.83 ± 0.15 (95.22)
P1' NH	A567: O	A572: O	2.78	3.02	---	3.02 ± 0.14 (85.05)
P1' CO	R738: NH2	R742: NH2	3.30	2.99	3.21 ± 0.18 (23.29)	3.04 ± 0.18 (8.71)
	R738: NH1	R742: NH1	3.20	3.68	---	3.18 ± 0.17 (5.83)
P2' NH	N566: OD1	N571: OD1	3.33	2.54	---	3.18 ± 0.18 (9.61)
P2' O2	N566: ND2	N571: ND2	2.49	2.55	---	2.93 ± 0.16 (95.05)
	R145: NH2	---	8.81	---	3.06 ± 0.21 (66.22)	---
P2' O3	R145: NH1	R723: NH1	6.78	6.56	2.86 ± 0.14 (97.37)	3.36 ± 0.13 (4.41)
	R145: NH2	R723: NH2	7.71	5.16	3.20 ± 0.20 (44.88)	2.88 ± 0.15 (98.51)
Indole NH	V565: O	L570: O	2.51	1.84	---	3.23 ± 0.18 (7.46)
			**Hydrophobic**			
P1' leucyl	I582: CD1	I587: CD1	3.30	3.77	3.82 ± 0.28	4.64 ± 0.42
P1' leucyl	V604: CG1	I609: CG2	4.30	3.56	4.15 ± 0.29	3.96 ± 0.24
P1' leucyl	W714: CH2	W719: CH2	4.32	3.39	3.71 ± 0.27	3.62 ± 0.20
P1' leucyl	F149: CE2	I150: CD1	3.80	4.48	---	---
P2' tryptophan	F149: CD2	I150: CG2	4.47	5.44	3.70 ± 0.32	---
P2' tryptophan	---	T146: CG2	---	2.70	---	3.85 ± 0.36

## Conclusions

The great interest in members of the M13 family of zinc peptidases as putative therapeutic targets and the discovery of new members of this family lead us to investigate the three-dimensional structure and dynamic flexibility of XCE (a novel member of the M13 family) and enable us to compare it with already well established member ECE-1. Homology modeling of XCE and docking of phosphoramidon into the modeled structure provided the primary information about its structural similarities and differences with ECE-1. Simulation of apo and phosphoramidon bound forms of XCE and ECE-1 revealed that the inhibitor induced distinct conformational changes surrounding the active site of both enzymes. The stability of I150, I609 and W719 of XCE and I582, V604 and W714 of ECE-1 suggest that these residues behave as the central determinant in the hydrophobic lock of their S1’ pockets. W714/W719 maintains 3–10 helical structure whereas R145/R723 and F149/T146 of ECE-1/XCE acquire antiparallel β-sheet structure for their strong binding to the inhibitor. Secondary structure changes of the S2’ residues (150–155) from α-helix to turn in aECE-1 explain the accomodation of large C-terminals of natural substrates into this region (endopeptidase activity) while their compactness in helical form clarify the difference in specificity of aXCE at this region. The carboxydipeptidase activity of ECE-1 is also justified by the folding of R145 into antiparallel β-sheet and similar role can be speculated for its structural counterpart R723 of XCE. Hence, larger variations and greater fluctuations in the S2’ subsite of both enzymes would be instrumental in determining their different substrate specificities. To the best of our knowledge, this is the first theoretical study of ECE-1 and XCE in context of their comparison. The structural features of both proteins and their dynamical behavior with phosphoramidon obtained from this study provide valuable information that can be used in the rational design of specific inhibitors for ECE-like proteins. However, molecular mechanism of substrate specificity in XCE needs to be ascertained carefully with the help of detailed experiments in the light of our preliminary theoretical study.

## Methods

### Homology modeling

The human XCE sequence (accession number O95672-1) [18] obtained from SWISS-PROT was searched for a suitable template using Blast [42] at NCBI database. As expected, the human ECE-1 phosphoramidon complex [31] was identified as the most befitting template with E-value of 2 × 10^−153^, thus exhibiting 39% identity and 60% similarity with the query sequence. The sequence of the ectodomain of human ECE-1 (90–770) was taken directly from Protein Data Bank and aligned to XCE sequence to compare the main features of both proteins by utilizing the program Jalview [43]. Homology modeling was carried out by utilizing the homology module implemented in the molecular operating environment (MOE) suite of programs [44]. MOE align facility was used to give the best alignment as input for modeling. Initially, 10 models were built and evaluated within the MOE package by a residue packing quality function, which depends on the number of buried nonpolar side-chain groups and on hydrogen bonding. Energy minimizations were carried out to remove geometrical strain using Amber99 potential [45] with RMS gradient of 0.01. The zinc ion and water molecule bound to zinc were positioned in a similar manner as observed in the ECE-1 structure by superimposing the homology model and template structure. The best intermediate model with the lowest energy was chosen for the quality assessment using Procheck [46].

### Molecular dynamics simulations

#### Starting structures for MD simulations

The apo form of both proteins denoted as aECE-1 and aXCE were constructed by coordinating the zinc ion to water molecule ‘Wat762’ and ‘Wat776’, respectively by using the zinc bound oxygen coordinates of phosphoramidon present in the pdb-id 3DWB (the complex of ECE-1 with the phosphoramidon inhibitor that was termed as cECE-1). The bioactive conformation of phosphoramidon was manually transferred into the modeled XCE to form the complexed form of XCE named as cXCE. Energy minimizations were carried out on the phosphoramidon and its interacting protein amino acids in cXCE to remove geometrical strain using Amber99 potential with the restraints applied on rest of the protein. To establish potentials for the complexed forms, electrostatic potential (ESP) for all atoms of phosphoramidon were calculated at HF level of theory using 6-31G* basis sets with Gaussian03 [47]. The ESP charges were then fitted to obtain restrained electrostatic potential (RESP) [48] charges with antechamber module of AMBER 10 [49]. It had been reported that the interaction energies between zinc and its coordinates were markedly underestimated by molecular mechanics calculations if the zinc ion was modeled octahedral traditionally [50]. To minimize the underestimation that would hamper the stability and interactions of zinc coordinates, the Cationic Dummy Atom (CaDA) approach was used. The approach contains four identical dummy atoms tetrahedrically attached to the zinc ion and transfers all the atomic charge of the zinc divalent cation evenly to the four dummy atoms [51]. The atoms are dummy in that sense they interact with other atoms electrostatically but not sterically, thus mimicking 4sp^3^ vacant orbitals of zinc atom accommodating its lone-pair electrons. The force field parameters of the tetrahedron-shaped zinc were used from the published protocol in molecular dynamics (MD) simulations [51,52]. In case of ECE-1, the first-shell zinc residues H607, H611, E667 and Wat762 while that of XCE, H612, H616, E672 and Wat776 were deprotonated as histidinate, glutamate, and hydroxide, respectively. In the second hydration shell of zinc ion, amino acids E608, D614, D671, H732 and E613, D619, D676, H737 were kept protonated in ECE-1 and XCE, respectively [53-56]. These structures were then solvated with cubic box of TIP3P water model extended to 8 Å beyond the surface of protein and neutralized with sodium ions. After preliminary modifications of all four systems, i.e. aXCE, cXCE, aECE-1, and cECE-1, each system was subjected to 10 ns molecular dynamics (MD) simulation using SANDER module of AMBER installed on a cluster computing facility at University of Karachi consisting 10 nodes.

#### Molecular dynamics simulation protocol

Force field parameters such as Duan *et al*. [57] and the generalized amber force field (gaff) [58] were utilized for protein and phosphoramidon, respectively. System topologies and coordinate files were generated with the xleap module of AMBER. Prior to MD simulations, stepwise energy minimizations were performed, which consist of 1000 steps on phosphoramidon with a positional constraint of 10 kcal mol^−1^ applied to rest of the complex. 50 steps energy minimizations on the tetrahedron shaped zinc divalent cation and another 50 steps energy minimizations on the zinc alongwith its coordinating residues and phosphoramidon were performed with a positional constraint applied to rest of the system followed by 2000 steps of unrestrained energy minimizations. Each system was gradually heated in the NPT ensemble from 0 to 300 K over 50 ps. All simulations used a dielectric constant of 1.0, a periodic boundary condition at a constant temperature of 300 K, a constant pressure of 1 atm with isotropic molecule based scaling and the SHAKE [59] algorithm applied to constrain all bonds involving the hydrogen atom. The temperature was rescaled with the Berendsen coupling algorithm [60] and the particle mesh ewald method [61,62] was used to calculate long range electrostatic interactions using a grid size of 80 × 96 × 90 (grid spacing of 1.0 Angstrom) combined with a fourth-order B-spline interpolation to compute the potential and forces in between grid points. Each system was equilibrated by gradually reducing the constraints and finally 10 ns simulation was performed with 2.0 fs time step. The resulting trajectories were analyzed using the PTRAJ included in AmberTools 1.4 package. Hydrogen bonds were defined to have a maximum hydrogen-acceptor distance of 3.5 Å and a minimum donor-hydrogen-acceptor angle of 120°. Hydrogen bonds possessing greater than 40% occupancy are classified as strong, 20–40% are weak, and less than 20% are termed as unstable over 10 ns simulation. Chimera [63] and VMD [64] were used to visualize the structures and trajectories obtained from MD simulation. Secondary structure analyses over 10 ns simulation were carried out employing the defined secondary structure of proteins (DSSP) method [65].

## Competing interests

The authors declare that they have no competing interests.

## Authors’ contributions

SI and STM designed and performed the experiments, and analyzed the data. SI wrote the paper and STM and Z-ul-H proof read the paper. All research has been done under the supervision of Z-ul-H. All authors read and approved the final manuscript.

## Supplementary Material

Additional file 1 **Figure S1.** The Ramachandran plot of the modelled XCE computed by procheck indicated reliable model building. Click here for file

Additional file 2 **Figure S2.** The Ramachandran plot of the equilibrated structure of apo XCE computed by procheck indicated reliable equilibration and relaxation. Click here for file

Additional file 3 **Figure S3.** The Ramachandran plot of the equilibrated structure of complexed XCE computed by procheck indicated reliable equilibration and relaxation. Click here for file

Additional file 4 **Figure S4.** The Ramachandran plot of the equilibrated structure of apo ECE-1 computed by procheck indicated reliable equilibration and relaxation. Click here for file

Additional file 5 **Figure S5.** The Ramachandran plot of the equilibrated structure of complexed ECE-1 computed by procheck indicated reliable equilibration and relaxation. Click here for file
