# Supplementary material for: Dynamic changes in the secondary structure of ECE-1 and XCE account for their different substrate specificities
Source: BMC Bioinformatics. 2012 Nov 1;13:285. doi: 10.1186/1471-2105-13-285 (PMC3558449; doi:10.1186/1471-2105-13-285)
Supplement: Additional file 1 — Figure S1. The Ramachandran plot of the modelled XCE computed by procheck indicated reliable model building. [file 1471-2105-13-285-S1.pdf]

PROCHECK

Ramachandran Plot

file

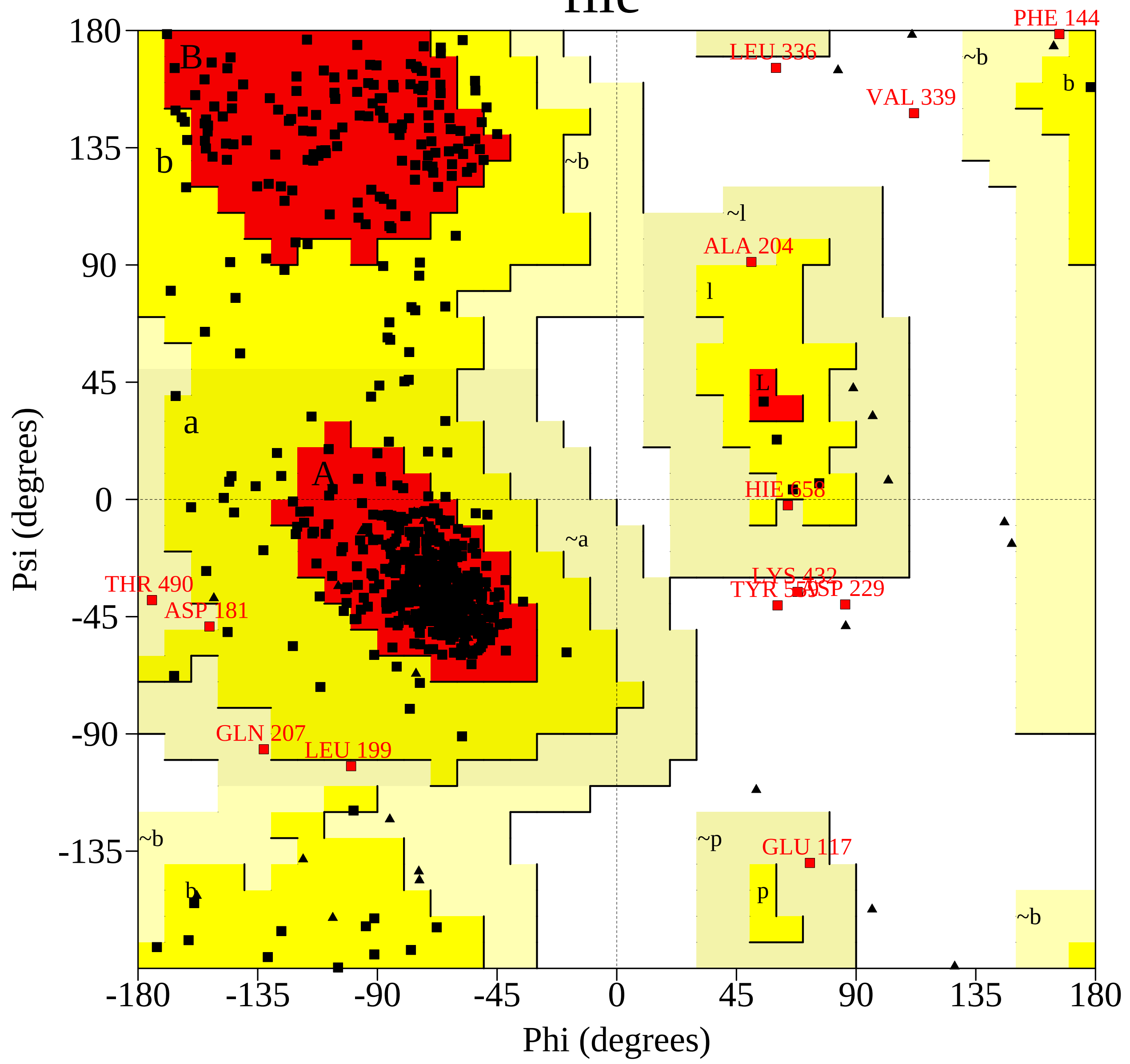

Plot statistics

Residues in most favoured regions [A,B,L]	507	84.6%
Residues in additional allowed regions [a,b,l,p]	79	13.2%
Residues in generously allowed regions [~a,~b,~l,~p]	8	1.3%
Residues in disallowed regions	5	0.8%
	----	------
Number of non-glycine and non-proline residues	599	100.0%
Number of end-residues (excl. Gly and Pro)	5	
Number of glycine residues (shown as triangles)	41	
Number of proline residues	29	

Total number of residues	674	

Based on an analysis of 118 structures of resolution of at least 2.0 Angstroms and R-factor no greater than 20%, a good quality model would be expected to have over 90% in the most favoured regions.

file_01.ps
